# Detecting Enteric Pathogens in Low-Risk Drinking Water in Dhaka, Bangladesh: An Assessment of the WHO Water Safety Categories

**DOI:** 10.3390/tropicalmed8060321

**Published:** 2023-06-14

**Authors:** Sabera Saima, Jannatul Ferdous, Rebeca Sultana, Ridwan Bin Rashid, Sara Almeida, Anowara Begum, Peter Kjær Mackie Jensen

**Affiliations:** 1 Department of Microbiology, University of Dhaka, 1000 Dhaka, Bangladesh; 2 Copenhagen Center for Disaster Research, Section for Global Health, Department of Public Health, University of Copenhagen, 1014 Copenhagen, Denmarkmackie@sund.ku.dk (P.K.M.J.); 3 Department of Life Sciences, School of Environment and Life Sciences, Independent University, 1229 Dhaka, Bangladesh; 4 icddr,b, 1212 Dhaka, Bangladesh; 5 Institute of Health Economics, University of Dhaka, 1000 Dhaka, Bangladesh

**Keywords:** drinking water, WHO guidelines, enteric pathogens, *E. coli*, Bangladesh

## Abstract

The microbiological quality of water is usually assessed by fecal coliform bacteria, and the presence of *E. coli* as an indicator of fecal contamination is widely recommended by international guidelines. This study aimed to assess the prevalence of diarrheagenic pathogens, in both public and personal domain water sources and examine the reliance on the WHO drinking water risk assessment guidelines. This study was conducted in a low-income urban community in Dhaka, Bangladesh between September 2014 and October 2015. Polymerase chain reaction (PCR) was used to detect the marker and virulence genes of *Escherichia coli*, *Vibrio cholerae*, *Salmonella* species, and *Campylobacter* species, and the culture method was employed for the quantitative assessment of *E. coli*. According to the WHO guidelines, 48% of the public domain source water and 21% of the personal domain point-of-drinking water were classified in the low-risk group, i.e., 0 CFU of *E. coli*/100 mL. However, when using PCR, we detected pathogens in 39% (14/36) of the point-of-drinking water samples and 65% (74/114) of the public domain water source samples classified in the low-risk group. Our study showed that relying solely on *E. coli* detection as a measure of water quality may overlook the presence of other pathogens in the drinking water. In addition to the culture-based method, the detection of virulence genes by PCR should also be considered to add more scrutiny to the detection of diverse types of pathogens.

## 1. Introduction

Globally, diarrhea remains a serious health problem, is recognized as the eighth leading cause of death, and is responsible for more than 1.57 million deaths annually across all age groups [1]. Unsafe water and sanitation were marked as the leading causes of diarrhea deaths (unsafe water 72.1% and unsafe sanitation 56.4%) in children younger than 5 years. These factors have a significant impact on the societal and individual household economy, particularly in lower-middle-income countries, such as Bangladesh [2,3]. 

The US Environmental Protection Agency (EPA), through its Candidate Contaminant List 3, has identified more than 500 bacterial pathogens of potential concern in drinking water on a global scale [4]. Subsequently, Ashbolt (2015) compiled a subset of reference pathogens containing the most critical enteric waterborne and water-based pathogens responsible for diarrheal diseases, namely *Escherichia coli (E. coli)*, *Vibrio cholerae (V. cholerae)*, *Salmonella* species, and *Campylobacter* species [5]. Of these, the predominant causative agent of bacterial diarrhea is diarrheagenic *Escherichia coli* (DEC), classified into enterotoxigenic *E. coli* (ETEC), enterohemorrhagic *E. coli* (EHEC), enteroinvasive *E. coli* (EIEC), enteroaggregative *E. coli* (EAEC), enteropathogenic *E. coli* (EPEC), and diffusely adhering *E. coli* (DAEC) [5,6]. ETEC, although requiring a high dose to cause infection, is included in the top 13 priority pathogens in the Global Burden of Disease (GBD) 2016 study list [6]. In 2016, ETEC and EPEC were responsible for approximately 51,000 and 12,000 deaths, respectively, across all age groups [2]. There are roughly 1.3 to 4.0 million cases and 21,000 to 143,000 deaths worldwide due to cholera caused by *V. cholerae* [6]. *Campylobacter jejuni* and *Campylobacter coli,* among all 17 described species of *Campylobacter* spp., are also associated with diarrheal diseases [7]. *Campylobacteriosis,* caused by *Campylobacter* spp., accounted for 8.4% of the total burden of diarrheal disease [8]. Worldwide, typhoid fever caused by *Salmonella* spp. (*Salmonella* serotype Typhi) affects approximately 21.6 million people and is responsible for 433,000 deaths every year [9]. All these pathogens can be transmitted to individuals by the fecal–oral route through contaminated water, food, and fomites [10,11,12].

The microbiological quality of water is usually assessed through the use of microbial indicators, such as the presence of fecal coliform bacteria. As such, the presence of *E. coli* as an indicator of fecal contamination is widely accepted. The World Health Organization (WHO) has developed a classification for the risk assessment of drinking water quality based on the quantitative number of fecal coliform bacteria in a culture-based assessment, with the presence of *E. coli* as an indicator of fecal contamination. According to the WHO drinking water risk assessment guidelines, water can be classified as Low risk/safe: no action required (<1 *E. coli*/100 mL); Intermediate risk: low action priority (1–10 *E. coli*/100 mL); High risk: higher action priority (11–100 *E. coli*/100 mL); and Very high risk: urgent/immediate action priority (>100 *E. coli*/100 mL) (WHO 2011) [13]. However, these risk assessment criteria concern contamination at the point of delivery in the public domain (at the public source) and not the quality inside the household, in the stored drinking water in the domestic domain at the ‘point-of-use’, or in the private domain at the ‘point-of-drinking’ (the definition of domains is provided in Jensen et al., 2023 [14]). Furthermore, the assessment only includes *E. coli* found by culture methods and does not account for *E. coli* found by non-culture methods or the presence of other pathogens. Therefore, this study aimed to assess the comparative prevalence, identified by culture and non-culture methods, of different diarrheagenic pathogens in water sources in the public domain and point-of-drinking water in the personal domain in low-income households in Bangladesh. In addition, this study aimed to evaluate whether the current microbiological method used in the WHO guidelines can serve as a proxy for pathogen exposure at the point-of-drinking in a low-income area in Dhaka, Bangladesh.

## 2. Materials and Methods

### 2.1. Study Design 

The study was conducted in East Arichpur, an urban area located northwest of the city of Dhaka, Bangladesh. The population density of Arichpur is high, with an approximate population of 55,500 living within a half km^2^ area. The study area was known to have a high incidence of cholera and waterborne diseases [15,16,17]. A research team experienced in qualitative and quantitative data collection conducted a series of activities, including a transect walk, informal group discussions, and participatory mapping, to enumerate all the households in the East Arichpur community. During this process, a census was conducted, resulting in a total of 13,876 households listed in the community. Within this locality, the cluster of households predominantly sharing poor housing, as well as shared water, sanitation, and/or cooking facilities were used as the inclusion criteria. These criteria were chosen to characterize and represent most of the low-income urban settings in Bangladesh [18]. The team selected a total of 477 households from the list of community households. The detailed methodology for selecting and recruiting these households is described in the protocol paper published by Sultana et al. in 2019 [17]. 

As part of routine visits at six-week intervals between September 2014 and October 2015, water samples were collected from both public and personal domains [18,19,20]. The water samples at the point of drinking were collected using the preferred drinking vessels, i.e., a mug, glass, bottle, jug, and pitcher, by the household members. In the public domain, the households used one of two types of groundwater-based water supply systems: a “WASA (Water Supply and Sewerage Authority) pump” installed by the municipal government and private submersible pumps (75–140 m) installed by individuals or groups of residents. The team collected 2514 point-of-drinking water samples and 1494 communal source water samples from September 2014 to October 2015 for basic water quality assessment [19]. For more in-depth analysis, a subsample of 409 water samples was randomly collected from point-of-drinking and public-sourced water: 169 samples were collected from 124 households at the point-of-drinking and 240 from 53 public domain sources. Detailed descriptions of the water supply infrastructure were provided by Ferdous et al. in 2021 [19]. 

### 2.2. Sample Collection and Processing

The water samples were collected using pre-sterilized wide-mouth water sampling bottles (SPL Life Sciences, Gyeonggi-do, Korea) and transported in a cool box to the Environmental Microbiology Laboratory at the University of Dhaka within 2–4 h of collection. Aliquots of 100 mL water samples were filtered through 0.45 µm 47 mm white gridded S-Pak Filters (Merck Millipore, Darmstadt, Germany) and the filters were placed on membrane thermotolerant *E. coli* agar (m-TEC agar, Oxoid, UK) plates. The plates containing the filters were incubated at 44.5 ± 0.5 °C for 18–24 h. After overnight incubation, typical reddish-purple or magenta colonies on the m-TEC were presumptively considered *E. coli* colonies and enumerated for CFU count of *E. coli* per 100 mL. The quality control of the membrane thermotolerant *E. coli* agar was analyzed whenever a new batch of media or reagents was used, following the USEPA method 1603 [21]. From each batch, one plate was incubated, and the absence of growth indicated media sterility. We performed media sterility checks every day.

For the detection of pathogenic bacteria, a 1 mL aliquot of the water samples was enriched in both alkaline peptone water (APW: 1 L distilled H_2_O, 10 gL^−1^ peptone, 10 gL^−1^ NaCl; pH 8.5) and nutrient broth (NB: 1 L distilled H_2_O, 1 gL^−1^ beef extract, 2 gL^−1^ yeast extract, 5 gL^−1^ NaCl). 

### 2.3. DNA Extraction and Detection of Virulence Gene

After overnight incubation at 37 °C, the genomic DNA was extracted from both the enriched APW and NB media using the boiled template method [22]. The extracted DNA was used as a template for the detection of the signature and pathogenic genes of *E. coli*, *V. cholerae, Campylobacter* spp., and *Salmonella* spp. through polymerase chain reaction (PCR). PCR was conducted using a Veriti™ 96-Well Fast Thermal Cycler (Thermo Fisher Scientific, Waltham, MA, USA). The 25-µL reaction mixture contained 2 µL of 10× PCR buffer, 20 mM MgCl_2_, 0.4 µL of 10 mM deoxynucleoside triphosphates (dNTP) mix (Thermo Scientific, Waltham, MA, USA), 0.1 µL of 5 U Dream Taq DNA Polymerase (Thermo Scientific, Waltham, MA, USA) per µL, and 1.25 µL of each 25 µM primer. The PCR reaction cycles were performed as follows: initial denaturation at 95 °C for 5 min followed by 95 °C for 1 min, 55 °C for 45 s, 72 °C for 45 s with 35 cycles including a final 7 min extension at 72 °C. To resolve the PCR products, 1.5% agarose gel in Tris-Acetate EDTA (TAE) buffer was used for electrophoretic separation. The gel was stained in Et-Br staining solution and observed under a UV transilluminator (Infinity Vilbert Loumart gel documentation system). The PCR product size was determined using 100 bp DNA size markers (Invitrogen, Waltham, MA, USA). The target genes, primer sequences, amplicon size, and amplification conditions are given in Table S1 (Supplementary Material) [23,24,25,26,27,28,29,30,31,32].

### 2.4. Data Analysis

All the descriptive statistics, such as the percentages, mean, and median with confidence intervals and interquartile ranges, were calculated using Microsoft Excel 2016.

## 3. Results

The households enrolled in the study used mugs, glasses, bottles, and jugs as point-of-drinking vessels for water. Among these drinking vessels, the mean fecal contamination was lower in bottled water (Table 1). Additionally, the mean fecal contamination was higher in treated drinking water than in untreated drinking water (Table 1). 

Among all the targeted diarrheagenic pathogens, the genes specific to diarrheagenic *E. coli* were the most prevalent and were detected in 37% (62/169) of the point-of-drinking water samples and 45% (109/240) of the public domain sourced water. The remaining pathogens accounted for 63% in the point-of-drinking water and 55% in the water sourced from the public domain (Table 2).

When stratifying the water samples by risk groups based on the initial *E. coli* count according to the WHO guidelines, pathogens were still found in the point-of-drinking water and public domain sourced water, even in the low-risk group with <1 CFU *E. coli*/100 mL. Among the other risk groups, the prevalence of pathogens was highest in the very high risk group for the point-of-drinking water samples (97%) and in the intermediate risk group for the public domain sourced water (74%) (Table 3). Additionally, the prevalence of the pathogens EIEC and EHEC, which require a very low dose to cause infection, was also higher in the low-risk group compared to the other risk groups.

Apart from single pathogens, multiple pathogens co-existing in drinking water were found in both the point-of-drinking and public-domain sourced water. Although the percentage was higher in the very high risk group for the point-of-drinking water, multiple pathogens co-existed in the low-risk group as well (Table 4). For the public domain sourced water, the scenario was reversed, where the low-risk group contained a high number of water samples that had multiple pathogens.

## 4. Discussion

In this study, we found the presence of pathogenic *E. coli* and other diarrheagenic bacteria through molecular methods (i.e., PCR) in water samples, while quantitative culture-based methods did not reveal the presence of detectable *E. coli* (1< *E. coli*/100 mL). The collected samples would have been classified into the low-risk group according to the WHO drinking water risk assessment guidelines, therefore stressing the safety of the WHO guidelines. Furthermore, the co-occurrence of multiple pathogens in the same samples was also detected. Apart from *E. coli*, *V. cholerae* was the most abundant pathogen.

Our results show that fecal contamination, along with the prevalence of diarrheagenic bacteria, were more frequent in the point-of-drinking water than in the public domain source water. Previous studies suggest that livestock ownership within household premises [33], the inappropriate washing and handling of utensils [34], and improper hand hygiene can affect the quality of drinking water. In our study area, we observed the presence of livestock in the compound area [17], and the detected *E. coli* isolates were found mainly to originate from animal sources [35]. Additionally, improper hygiene conditions, such as the presence of *E. coli* on kitchen utensils [36], could be linked to the higher contamination levels in the point-of-drinking water. The higher fecal contamination in the point-of-drinking water compared to the source water may be attributed to post-treatment contamination within households resulting from the improper handling of treated water, such as contaminated hands, kitchen utensils [37], dishwashing areas [38], and floors [39]. Improving kitchen hygiene practices (e.g., hand hygiene, covering kitchen pans, washing dirty vessels, etc.) and the careful management of post-treated water may help reduce post-contamination [40].

Among the identified *E. coli* pathotypes, ETEC was the most prevalent in drinking water, followed by aEPEC, EIEC, EHEC, and EAEC. The prevalence of ETEC was 14% (24/169) in the ‘point-of-drinking’ water and 18% (44/240) in the public domain source water. Moreover, the cumulative proportion of aEPEC, EIEC, EHEC, and EAEC pathotypes was 22% (38/169) in the ‘point-of-drinking’ water and 27% (65/240) in the public domain source water. This is particularly concerning as the presence of aEPEC, EIEC, EHEC, and EAEC in drinking water can cause severe infection, even at extremely low doses, especially for EIEC and EHEC (1 to 100 organisms) [41,42,43]. A study conducted by Ferdous et al. in 2021 also reported similar results regarding the dominant pathotype ETEC in the same study area, where isolates were collected using conventional culture-based methods [35].

After *E. coli,* the second most prevalent pathogen detected in the drinking water was *V. cholerae,* followed by *Salmonella* spp. and *Campylobacter* spp. *V. cholerae* is the etiologic agent of cholera, which is endemic in Bangladesh. The presence of both toxigenic and nontoxigenic *V. cholerae* is of public health concern as they have been associated with sporadic cases and outbreaks of cholera-like disease [44,45]. Although *Salmonella* spp. and *Campylobacter* spp. were less abundantly detected in the samples, there have been reports of outbreaks caused by these pathogens in drinking water [46,47]. Any detection of *Salmonella* spp. and *Campylobacter* spp. in drinking water is alarming due to their low infectious dose, which can be as low as 10 cells for *Salmonella* spp. [48] and ca. 100 cells for *Campylobacter jejuni* [49]. 

In summary, pathogens were detected in samples in the low-risk group (<1 *E. coli*/100 mL), both in the ‘point-of-drinking’ and source water samples. In the public domain source water, more pathogens were detected in samples that were classified in the low-risk group rather than in any of the other WHO higher-risk groups. However, the relationship between microbial water quality and diarrhea is still not completely clear. In 1991, Moe et al. [50] found that there was no association between the threshold effect and increased diarrheal disease up to 1000 *E. coli*/mL, and in 2001, Jensen [51] did not find any association at all. Conversely, Luby et al. in 2015 [52] found that each 10-fold increase in *E. coli* contamination in drinking water was associated with a 16% increase in diarrhea. However, these studies based their conclusions on the investigation of coliforms and indicator organisms, such as *E. coli,* using the culture method and did not investigate the presence of other pathogenic organisms. In our study, the presence of pathogenic organisms in the low-risk group indicates that the use of the *E. coli* CFU count as an indicator of pathogen presence can be problematic. Our study suggests that relying solely on the bacterial CFU count for water quality testing would be precarious, and, thus, the presence of diarrheagenic bacteria in drinking water should be investigated. The WHO guidelines that categorize risk assessment based on the CFU count could be misleading if the low-risk category is considered safe for drinking, as the presence of low/no detectable *E. coli* (0 CFU/100 mL) does not strictly imply the absence of any diarrheagenic gene-carrying pathogens. 

Our findings on the presence of diarrheagenic pathogens in low-risk groups could be an indication of the occurrence of bacteria in the viable but non-culturable (VBNC) state. Previous studies showed that *E. coli*, *V. cholerae*, *Salmonella* spp., and *Campylobacter* spp. can all exist in a dormant VBNC state after prolonged exposure in water, e.g., *E. coli* up to 100 days [53,54], *Salmonella* spp. 4 months in a water microcosm [55], and *V. cholerae* > 700 days [56]. In our study, despite no detection of *E. coli* by the culture method, 20.8% (50/240) of the source water samples and 6.5% (11/169) of the point-of-drinking water samples in the low-risk group were found to be positive for pathogens carrying diarrheagenic genes. This is consistent with a study in Isfahan, Iran where samples were positive for both *E. coli* and *Salmonella* spp. by PCR, but no bacterial colonies were detected by the culture method [57]. To accommodate the possibility of VBNC cells, it is advised to employ different methods in water quality testing and correlate the results to accurately detect the true extent of the contamination.

## 5. Limitations

One limitation of our study is the use of PCR for the detection of virulence genes, which cannot differentiate between dead and viable cells and might lead to an overestimation. However, there are reports of the natural transformability of *E. coli* in water [58,59,60]. This suggests that the bacterial DNA of dead cells existing in an environment such as water could be taken up by competent *E. coli*, thereby perpetuating infection. Furthermore, when there is extracellular DNA (exDNA), which is released due to cell lysis and death, high bacterial abundance accelerates the degradation of exDNA, even within 24 h [61], indicating that DNA from dead cells does not exist for long when there is an abundance of bacteria. Therefore, the presence of pathogenic genes in the drinking water in our study, even if originating from dead cells, could be alarming, suggesting recent bacterial degradation or uptake by competent cells. Another limitation of our study is that we did not measure the concentration of the pathogenic bacteria. Measuring the concentration of the pathogenic bacteria would have strengthened this paper by providing an understanding of the dose responsible for causing infection. However, including this concentration of pathogens in future studies would be informative for assessing health risks related to pathogen exposure. 

## 6. Conclusions

Most of the studies on water quality, including those conducted by Luby et al. (2015), Wardrop et al. (2018), Amin et al. (2019), and Luoto et al. (2011) [33,52,62,63], have primarily focused on the presence of *E. coli* in drinking water, which may be suboptimal for detecting the presence of diarrheagenic organisms. Our study findings showed that relying solely on *E. coli* as a measure of water quality may lead to overlooking the presence of other pathogens in drinking water. In addition to the culture-based method, the detection of virulence genes by PCR should also be considered to enhance the identification and detection of diverse types of pathogens. Furthermore, our study revealed a higher proportion of pathogens at the point-of-drinking when compared to source water, indicating that more focus should be given to the personal domain (e.g., point-of-drinking water) to prevent contamination within the household.

## Figures and Tables

**Table 1:** The mean and median value of *E. coli* by culture-based method in point-of-drinking water and public source water of the study households, stratified by various characteristics.

Characteristics	Sample N (%)	Mean (CI) *E. coli* CFU/100 mL	Median (IQR1, IQR3) *E. coli* CFU/100 mL
**Public domain source water [N = 240]**			
WASA	23 (10)	24 (6, 43)	0 (0, 30)
Submersible pumps	217 (90)	62 (45, 80)	4 (0, 69)
**Point-of-drinking water [N = 169]**			
Treated water	28 (16)	106 (45, 167)	26 (4, 203)
Non-treated water	142 (84)	63 (45, 81)	12 (1, 61)
**Types of treatment carried out in the households**			
Boiling	21 (12)	111 (29, 193)	22 (4, 194)
Filtration	7 (4)	101 (−5, 206)	28 (4, 208)
**Types of drinking vessels used at the point-of-drinking**			
Mug	82 (49)	85 (56, 114)	24 (4, 113)
Glass	53 (31)	78 (43, 113)	16 (0.5, 99)
Bottle	28 (17)	19 (5, 33)	8 (0.5, 22)
Jug	6 (3)	41 (−48, 129)	4 (2, 68)

**Table 2:** Distribution of diarrheagenic pathogens using PCR method, in public domain source and ‘point-of-drinking’ water.

Pathogen	Genes	Point-of-Drinking Water, N = 169 (%)	Public Domain Source Water, N = 240 (%)
ETEC	*eltB*	4 (2)	15 (6)
	*estA*	20 (12)	24 (10)
	*eltB* + *estA*	0 (0)	5 (2)
EHEC	*vt1*	7 (4)	4 (2)
	*vt2*	6 (4)	5 (2)
	*vt2* + *eaeA*	1 (1)	1 (0.4)
	*vt1 + vt2 + eaeA*	1 (1)	0
aEPEC *	*bfpA*	2 (1)	29 (12)
	*eaeA*	10 (6)	3 (1)
EIEC	*ial*	1 (1)	13 (5)
	*ipaH*	9 (5)	5 (2)
EAEC	pCVD	1 (1)	5 (2)
*Vibrio cholerae*	*ompW*	26 (15)	26 (11)
*Salmonella* spp.	*invA*	3 (2)	6 (3)
*Salmonella enteritidis*	IE-1	0	1 (0.4)
*Salmonella typhimurium*	flic-C	2 (1)	3 (1)
*Campylobacter* spp.	16srRNA	4 (2)	2 (1)
*Campylobacter coli*	cueE	3 (2)	0
*Campylobacter jejuni*	cj0414	0	1 (0.4)
Total pathogen		100 (59)	148 (62)

* aEPEC-atypical EPEC.

**Table 3:** Distribution of diarrheagenic pathogens in WHO risk groups categorized by CFU of *E. coli*/100 mL for ‘point-of-drinking’ water and public domain source water samples.

Risk Group	Point of Collection	ETEC	EHEC	aEPEC	EIEC	EAEC	*V. cholerae*	*Salmonella* spp.	*Campylobacter* spp.	Total Pathogens (%)
**Low risk** **(<1 *E. coli*/100 mL)**	Point-of- drinking, N = 36 (%)	2 (6)	5 (14)	2 (6)	0	0	4 (11)	0	1 (3)	14 (39)
	Public domain source, N = 114 (%)	26 (23)	6 (5)	20 (18)	10 (9)	2 (2)	5 (4)	4 (4)	1 (0.9)	74 (65)
	Total N = 150	28 (19)	11 (7)	22 (15)	10 (7)	2 (1)	9 (6)	4 (3)	2 (1)	88 (59)
**Intermediate risk** **(1–10 *E. coli*/100 mL)**	Point-of- drinking, N = 35 (%)	3 (9)	0	5 (14)	1 (3)	1 (3)	2 (6)	0	0	12 (34)
	Public domain source, N = 23 (%)	5 (22)	0	4 (17)	2 (3)	1 (4)	5 (22)	0	0	17 (74)
	Total N = 58	8 (14)	0	9 (16)	3 (5)	2 (3)	7 (12)	0	0	29 (50)
**High risk** **(11–100 *E. coli*/100 mL)**	Point-of- drinking, N = 61 (%)	7 (11)	5 (8)	4 (7)	4 (7)	0	9 (15)	2 (3)	2 (3)	33 (54)
	Public domain source, N = 61 (%)	8 (13)	1 (2)	8 (13)	3 (5)	1 (2)	9 (15)	2 (3)	1 (2)	33 (54)
	Total N = 122	15 (12)	6 (5)	12 (10)	7 (6)	1 (1)	18 (15)	4 (3)	3 (2)	66 (54)
**Very High risk** **(>100 *E. coli*/100 mL)**	Point-of- drinking, N = 37 (%)	12 (32)	5 (14)	1 (3)	5 (14)	0	11 (30)	1 (3)	1 (3)	36 (97)
	Public domain source, N = 42 (%)	5 (12)	3 (7)	0	3 (7)	1 (2)	7 (17)	0	0	19 (45)
	Total N = 79	17 (22)	8 (10)	1 (1)	8 (10)	(1)	18 (23)	1 (1)	1 (1)	55 (70)

**Table 4:** Distribution of ‘point-of-drinking’ and public domain source water samples contaminated with single and multiple pathogens in different WHO risk groups.

	Low Risk (<1 *E. coli*/100 mL)		Intermediate Risk (1–10 *E. coli*/100 mL)		High Risk (11–100 *E. coli*/100 mL)		Very High Risk (>100 *E. coli*/100 mL)	
***Point-of-drinking***								
Single pathogen, *N (%)*	8 (22%)		4 (11%)		21 (34%)		17 (46%)	
Co-existing pathogens > 1, *N (%)*	3 (8%)		4 (11%)		6 (10%)		9 (24%)	
List of co-existing pathogens, N	ETEC + EHEC	1	ETEC + aEPEC	1	ETEC + EHEC	2	ETEC + EHEC	1
	ETEC + *V. cholerae*	1	EPEC + EAEC	1	ETEC + EIEC	1	ETEC + *V. cholerae*	3
	EHEC + *V. cholerae*	1	aEPEC + *V. cholerae*	2	ETEC + *V. cholerae*	1	EHEC + *V. cholerae*	2
					*V.cholerae+ Salmonella typhi*	1	EIEC +*V. cholerae*	1
					EPEC + *V. cholerae*	1	ETEC + EIEC	1
							ETEC + EIEC + *V. cholerae*	1
***Public domain source***								
Single pathogen, *N (%)*	30 (28%)		8 (35%)		15 (25%)		11 (26%)	
Co-existing pathogens > 1, *N (%)*	20 (17%)		4 (17%)		8 (13%)		4 (10%)	
List of co-existing pathogens, N	EPEC + EIEC	3	ETEC + EPEC	1	ETEC + EAEC	1	ETEC + EAEC	1
	ETEC + EPEC	4	ETEC + EAEC + EIEC	1	ETEC + EIEC	1	ETEC + EIEC	1
	ETEC + EAEC	1	EPEC + *V. cholerae*	2	ETEC + EPEC	2	EPEC + *V. cholerae*	1
	EHEC + EPEC	2			ETEC + EPEC + EIEC + *V. cholerae*	1	EIEC + *V. cholerae*	1
	ETEC + EHEC	1			EPEC + *V. cholerae*	2		
	ETEC + EIEC	2			ETEC + *V. cholerae*	1		
	ETEC + EHEC+ EPEC	1						
	ETEC + EPEC + EIEC	1						
	EPEC + EIEC + *V. cholerae*	1						
	ETEC + EPEC + *Salmonellae enteritidis*	1						
	ETEC + *Salmonella typhimurium*	1						
	ETEC + *Salmonella enteritidis*	1						
	*V.cholerae + Campylobacter coli*	1						

## Data Availability

The data are contained within the article.

## Supplementary Materials

The following supporting information can be downloaded at https://www.mdpi.com/article/10.3390/tropicalmed8060321/s1, Table S1: Target genes, primer sequences, amplicon size for detection of the signature and pathogenic genes of bacteria.
