# Supplementary material for: Detecting Enteric Pathogens in Low-Risk Drinking Water in Dhaka, Bangladesh: An Assessment of the WHO Water Safety Categories
Source: Trop Med Infect Dis. 2023 Jun 14;8(6):321. doi: 10.3390/tropicalmed8060321 (PMC10302631; doi:10.3390/tropicalmed8060321)
Supplement: Supplementary file 1 [file tropicalmed-08-00321-s001.zip › tropicalmed-2236717-supplementary.pdf]

Tropical Medicine and Infectious Disease

MDPI

# Supplementary Materials of Detecting Enteric Pathogens in Low-Risk Drinking Water in Dhaka, Bangladesh: An Assessment of the WHO Water Safety Categories

**Table S1.** Target genes, primer sequences, amplicon size for detection of the signature and pathogenic genes of bacteria.

| Genes | Primer Name | Primer Sequences (5′→ 3′) | Amplicon Size (bp) | References |
|---|---|---|---|---|
| | | Diarrheagenic *E. coli* genes | | |
| *eltB* | LT-F | tctctatgtgcatacggagc | 322 | [1] |
| | LT-R | ccatactgattgccgcaat | | |
| *estA* | ST-F | gctaaaccagta$^{g}_{a}$ggtcttcaaaa | 147 | [1] |
| | ST-F | cccggtaca$^{g}_{a}$gcaggattacaaca | | |
| *vt1* | VT1-F | gaagagtccgtgggattacg | 130 | [2] |
| | VT1-R | agcgatgcagctattaataa | | |
| *vt2* | VT2-F | accgtttttcagatttt$^{g}_{a}$cacata | 298 | [1] |
| | VT2-R | tacacaggagcagtttcagacagt | | |
| *eaeA* | eae-F | cacacgaataaactgactaaaatg | 376 | [1] |
| | eae-R | aaaaacgctgacccgcacctaaat | | |
| *bfpA* | bfpA-F | ttcttggtgcttgcgtgtctttt | 367 | [1] |
| | bfpA-R | ttttgtttgttgtatctttgtaa | | |
| *ipaH* | ipaH-F | gctggaaaaactcagtgcct | 424 | [3] |
| | ipaH-R | ccagtccgtaaattcattct | | |
| pCVD | EA-F | ctggcgaaagactgtatcat | 630 | [4] |
| | EA-R | caatgtatagaaatccgctgtt | | |
| | | *V. cholerae* species specific gene | | |
| *ompW* | ompW-F | caccaagaaggtgactttattgtg | 304 | [5] |
| | ompW-R | ggtttgtcgaattagcttcacc | | |
| | | *Salmonella* spp. | | |
| *invA* | S-invA-F | cggtggttttaagcgtactctt | 796 | [6] |
| | S-invA-R | cgaatatgctccacaaggtta | | |
| | | *Salmonella enteritidis* | | |
| *IE-1* | SE-IE1-F | agtgccatacttttaatgac | 316 | [7] |
| | SE-IE1-R | actatgtcgatacggtggg | | |
| | | *Salmonella typhimurium* | | |
| *Flic-C* | ST-flic C-F | cccgcttacaggtggactac | 432 | [8] |
| | ST-flic C-R | agcgggttttcggtggttgt | | |
| | | *Campylobacter* spp. | | |
| 16S rRNA | C412F | ggatgacacttttcggagc | 816 | [9] |
| | C1228R | cattgtagcacgtgtgtc | | |
| | | *Campylobacter coli* | | |
| *cueE* | CC18F | ggtatgatttctacaaagcgag | 502 | [9] |
| | CC519R | ataaaagactatcgtcgcgtg | | |
| | | *Campylobacter jejuni* | | |
| cj0414 | CJ-F | caaataaagttagaggtagaatgt | 161 | [10] |
| | CJ-R | ccataagcactagctagctgat | | |

## References


1. Svenungsson, B.; Lagergren, Å.; Ekwall, E.; Evengård, B.; Hedlund, K.O.; Kärnell, A.; Löfdahl, S.; Svensson, L.; Weintraub, A. Enteropathogens in adult patients with diarrhea and healthy control subjects: A 1-year prospective study in a Swedish clinic for infectious diseases. *Clin. Infect. Dis.* **2000**, *30*, 770–778.
2. Pollard, D.; Johnson, W.; Lior, H.; Tyler, S.; Rozee, K. Rapid and specific detection of verotoxin genes in Escherichia coli by the polymerase chain reaction. *J. Clin. Microbiol.* **1990**, *28*, 540–545.
3. Tornieporth, N.G.; John, J.; Salgado, K.; de Jesus, P.; Latham, E.; Melo, M.; Gunzburg, S.T.; Riley, L.W. Differentiation of pathogenic Escherichia coli strains in Brazilian children by PCR. *J. Clin. Microbiol.* **1995**, *33*, 1371–1374.
4. Schmidt, H.; Knop, C.; Franke, S.; Aleksic, S.; Heesemann, J.; Karch, H. Development of PCR for screening of enteroaggregative Escherichia coli. *J. Clin. Microbiol.* **1995**, *33*, 701–705.
5. Nandi, B.; Nandy, R.K.; Mukhopadhyay, S.; Nair, G.B.; Shimada, T.; Ghose, A.C. Rapid method for species-specific identification of Vibrio cholerae using primers targeted to the gene of outer membrane protein OmpW. *J. Clin. Microbiol.* **2000**, *38*, 4145–4151.
6. Fratamico, P.M. Comparison of culture, polymerase chain reaction (PCR), TaqMan Salmonella, and Transia Card Salmonella assays for detection of Salmonella spp. in naturally-contaminated ground chicken, ground turkey, and ground beef. *Mol. Cell. Probes* **2003**, *17*, 215–221.
7. Wang, S.J.; Yeh, D.B. Designing of polymerase chain reaction primers for the detection of Salmonella enteritidis in foods and faecal samples. *Lett. Appl. Microbiol.* **2002**, *34*, 422–427.
8. Paião, F.; Arisitides, L.; Murate, L.; Vilas-Bôas, G.; Vilas-Boas, L.; Shimokomaki, M. Detection of Salmonella spp, Salmonella Enteritidis and Typhimurium in naturally infected broiler chickens by a multiplex PCR-based assay. *Braz. J. Microbiol.* **2013**, *44*, 37–42.
9. Linton, D.; Lawson, A.; Owen, R.; Stanley, J. PCR detection, identification to species level, and fingerprinting of Campylobacter jejuni and Campylobacter coli direct from diarrheic samples. *J. Clin. Microbiol.* **1997**, *35*, 2568–2572.
10. Wang, R.F.; Slavik, M.F.; Cao, W.W. A Rapid PCR Method for Direct Detection of Low Numbers of Campylobacter Jejuni. *J. Rapid Methods Autom. Microbiol.* **1992**, *1*, 101–108.


**Disclaimer/Publisher's Note:** The statements, opinions and data contained in all publications are solely those of the individual author(s) and contributor(s) and not of MDPI and/or the editor(s). MDPI and/or the editor(s) disclaim responsibility for any injury to people or property resulting from any ideas, methods, instructions or products referred to in the content.
